# Recombinant Slit2 suppresses neuroinflammation and Cdc42-mediated brain infiltration of peripheral immune cells via Robo1–srGAP1 pathway in a rat model of germinal matrix hemorrhage

**DOI:** 10.1186/s12974-023-02935-2

**Published:** 2023-10-29

**Authors:** Qian Li, Lei Huang, Yan Ding, Prativa Sherchan, Wenjie Peng, John H. Zhang

**Affiliations:** 1 Department of Pediatrics, Army Medical Center, Army Medical University, 10 Changjiang Access Rd, Yuzhong District, Chongqing, 400042 China; 2 Women and Children’s Hospital of Chongqing Medical University, 120 Longshan Access Rd, Yubei District, Chongqing, 400010 China; 3 Department of Physiology and Pharmacology, School of Medicine, Loma Linda University, 11041 Campus Street, Loma Linda, CA 92354 USA; 4 Department of Neurosurgery, School of Medicine, Loma Linda University, 11234 Anderson Street, Loma Linda, CA 92354 USA

**Keywords:** Germinal matrix hemorrhage, Slit2, Robo1–srGAP1, Neuroinflammation, Apoptosis, Rat

## Abstract

**Background:**

Germinal matrix hemorrhage (GMH) is a devastating neonatal stroke, in which neuroinflammation is a critical pathological contributor. Slit2, a secreted extracellular matrix protein, plays a repulsive role in axon guidance and leukocyte chemotaxis via the roundabout1 (Robo1) receptor. This study aimed to explore effects of recombinant Slit2 on neuroinflammation and the underlying mechanism in a rat model of GMH.

**Methods:**

GMH was induced by stereotactically infusing 0.3 U of bacterial collagenase into the germinal matrix of 7-day-old Sprague Dawley rats. Recombinant Slit2 or its vehicle was administered intranasally at 1 h after GMH and daily for 3 consecutive days. A decoy receptor recombinant Robo1 was co-administered with recombinant Slit2 after GMH. Slit2 siRNA, srGAP1 siRNA or the scrambled sequences were administered intracerebroventricularly 24 h before GMH. Neurobehavior, brain water content, Western blotting, immunofluorescence staining and Cdc42 activity assays were performed.

**Results:**

The endogenous brain Slit2 and Robo1 expressions were increased after GMH. Robo1 was expressed on neuron, astrocytes and infiltrated peripheral immune cells in the brain. Endogenous Slit2 knockdown by Slit2 siRNA exacerbated brain edema and neurological deficits following GMH. Recombinant Slit2 (rSlit2) reduced neurological deficits, proinflammatory cytokines, intercellular adhesion molecules, peripheral immune cell markers, neuronal apoptosis and Cdc42 activity in the brain tissue after GMH. The anti-neuroinflammation effects were reversed by recombinant Robo1 co-administration or srGAP1 siRNA.

**Conclusions:**

Recombinant Slit2 reduced neuroinflammation and neuron apoptosis after GMH. Its anti-neuroinflammation effects by suppressing onCdc42-mediated brain peripheral immune cells infiltration was at least in part via Robo1–srGAP1 pathway. These results imply that recombinant Slit2 may have potentials as a therapeutic option for neonatal brain injuries.

**Supplementary Information:**

The online version contains supplementary material available at 10.1186/s12974-023-02935-2.

## Introduction

Germinal matrix hemorrhage (GMH) occurs while fragile blood vessels are ruptured inside the subependymal brain tissue [1]. Preterm infants are the most vulnerable group suffering from GMH, with approximate 3.5 cases out of every 1000 births. GMH leads to developmental delays, mental retardation, cerebral palsy, and even posthemorrhagic hydrocephalus with devastating effects [1, 2]. There are two levels of brain damage caused by GMH. The primary damage takes place within the first few hours after hemorrhage and is largely due to hematoma formation, which causes mechanical harm to adjoining tissues [3]. Secondary brain damage causes the further neurological deterioration with significant inflammatory responses [4]. As current treatments for GMH-induced primary damage have not shown conclusive benefits in clinical trials yet, vast majority of studies are focusing on secondary damage to find effective therapeutic targets [4–6].

Several studies have shown that pronounced inflammatory responses occur during GMH-induced secondary brain injury [7–9]. Following brain hemorrhage, resident immune cells are activated and release proinflammatory cytokines and chemokines, such as tumor necrosis factor-α (TNF-α) and interleukin 6 (IL-6), inducing endothelial cells to upregulate intercellular adhesion molecule (ICAM-1) [10, 11]. Such proinflammatory signaling also promotes the migration of peripheral immune cells into the brain that further exacerbate neuroinflammation after GMH [12–16].

Slit family are secreted extracellular matrix-associated glycoproteins, which inhibit leukocyte chemotaxis, regulate axon guidance and control neuronal migration. They are expressed endogenously on neurons and astrocytes in the brain [17, 18]. Vascular endothelial cells secrete Slit2 binds to the transmembrane receptor Robo1 on leukocytes and acts as an endogenous inhibitor of leukocyte chemotaxis [17, 19, 20]. The Slit2 signaling has been shown to be protective in experimental models of systemic inflammation [21–23]. The systemic administration of Slit2 inhibited leukocyte migration to cortical venules after the global cerebral ischemia in mice [24].

Slit2 regulates neuron migration by enhancing the interaction between the Robo1 receptor and Slit-RoboGTPase-activating protein 1 (srGAP1), a downstream effector of Slit2/Robo1signaling [25–27]. The small Rho GTPase Cdc42 [28], a critical mediator of cell motility and neuronal guidance, is subsequently inactivated upon the activation of Slit signaling [20, 29–31]. These genes are also expressed in leukocytes[29]. Robo1 was expressed on the surface of peripheral immune cells [26]. Therefore, the Robo1/srGAP1/Cdc42 signal transduction pathway mediates the anti-migratory effect of Slit2, which may attenuate the peripheral immune cells infiltration into the brain.

Our previous studies demonstrated the protective effects of Slit2 on blood brain barrier (BBB) preservation and anti-neuroinflammation in an adult rat model of surgical brain injury [26]. Given the differences in pathophysiology and age population of GMH, the role of Slit2 remains unclear in in the setting of GMH. This study aimed to evaluate the effects of recombinant Slit2 as a potential treatment to minimize neuroinflammation, and to explore potential underlying mechanisms of Robo1/srGAP1 in inhibiting Cdc42-mediated brain infiltration of peripheral immune cells after the experimental GMH.

## Materials and methods

All procedures are conducted in accordance with the National Institutes of Health Guidelines for Laboratory Animal Handling and are approved by the Institutional Animal Care and Use Committee at Loma Linda University. A total of 240 Sprague–Dawley rat pups aged of 7 days old (P7) were used in this study. The brain development stage of P7 in a rat is comparable to that of a infant between 30 and 32 weeks of gestation.

### Experimental design and groups

#### Experiment 1

To determine the time course of endogenous brain Slit2 and Robo1 expression following GMH, the P7 rat pups (*n* = 42, 6/group) were randomly divided into five groups: sham, GMH day 1, GMH day 3, GMH day 5, and GMH day 7. Whole brain tissues were used for Western blotting. Additional 12 rats from groups of sham (6/group), GMH day 1 (3/group) and day 5 (3/group) were used for immunofluorescence staining.

#### Experiment 2

To determine whether knockdown of endogenous Slit2 exacerbated brain edema, thereby worsening short-term neurological deficits after GMH, the P7 rat pups were randomized to the following experimental groups: sham, GMH, GMH + Slit2 siRNA, and GMH + Scramble siRNA. Brain water content (*n* = 24, 6/group) was evaluated at 24 h after GMH. Another set of animals (*n* = 24, 6/group) were used to evaluate the short-term neurological deficits from 1 to 3 days after GMH, after which animals were euthanized for Western blotting on day 5 after GMH.

#### Experiment 3

The effects of exogenous recombinant Slit2 treatment on GMH were evaluated. Three doses of recombinant Slit2 (1, 3, and 10 μg/kg, R and D Systems, Minneapolis, MN, USA) were tested. Dosage was selected based on the dose–response effect of Slit2 on T cell chemotaxis previously reported in in vitro study [30] and we examined the effects of two additional doses to determine the optimal dose for GMH. The P7 rat pups (*n* = 30, 6/group) were randomly divided into five groups: sham, GMH + vehicle, GMH + Slit2 (1 μg/kg), GMH + Slit2 (3 μg/kg), and GMH + Slit2 (10 μg/kg). Short-term neurological assessment was performed during 1–3 days after GMH and brain samples were collected for Western blot analysis on day 5. Additionally, another set of rats were randomly divided into three groups: sham, GMH + Vehicle, and GMH + Slit2 (10 μg/kg), in which Western blotting was performed to validate the delivery efficiency of intranasal administration of Slit2 (10 μg/kg, *n* = 12, 4/group) and apoptosis marker cleaved caspase 3 (*n* = 24, 6/group); TUNEL staining (*n* = 18, 3/group) was used to assess neuron death.

#### Experiment 4

The role of Robo1 and srGAP1 in recombinant Slit2-mediated protection after GMH was investigated. Rats pups (*n* = 54, 6/group) were randomly divided into six groups: sham, GMH + vehicle, GMH + Slit2 (10 μg/kg), GMH + Slit2 (10 μg/kg) + Robo1 (3 μg/kg), GMH + Slit2 (10 μg/kg) + srGAP1 siRNA, and GMH + Slit2 (10 μg/kg) + scrambled siRNA, of which a set of 36 rats were used for long-term neurobehavioral evaluation and another set of 18 from last three groups with shared brain sampled from experiment 2 were used for Western bloting and Cdc42 activity assays. Recombinant Robo1 (3 μg/kg) (R&D Systems, Minneapolis, MN, USA) was co-administered with recombinant Slit2 by intranasal route at 1 h after GMH and daily for 3 consecutive days. srGAP1 siRNA or scrambled siRNA (Life Technologies, Grand Island, NY, USA) was administered via intracerebroventricular (i.c.v.) injection at 24 h before GMH. Brain samples were collected on day 5 for Western blotting and Cdc42 activity assay. Long-term neurological function was evaluated between 21 and 28 days after GMH.

### GMH model and treatments

As previously described, we induced grade III or IV GMH by stereotactic infusion of 0.3U bacterial collagenase into the right ganglion bulge of rat pup [31]. In short, the rat pups were anesthetized with 3% isoflurane (mixed air and oxygen) and placed on a stereotactic headframe. All animal operations were performed using aseptic techniques. The anterior horn was found after a small incision in the longitudinal midline of the scalp. With respect to the front corner, a 1-mm burr hole was formed at 1.6 mm on the nozzle side and 1.5 mm on the right side. The No. 27 needle was inserted at the depth of 2.8 mm from the burr hole in a stereotactic manner and collagenase (1 μl, 0.3 U/μL) was injected (1 μL/min). The needle stayed in place for another 5 min to prevent collagenase from flowing back along the needle path. After the syringe was withdrawn, the burr hole was sealed with bone wax and the incision was closed with suture. The sham animals received the same procedure, except for the infusion of collagenase.

Recombinant Slit2 (6μL; 1, 3, and 10 μg/kg) or vehicle (phosphate-buffered saline, PBS) was administered intranasally at 1 h and daily for 3 consecutive days after GMH induction. Recombinant Robo1, used as a decoy receptor, was co-administered with recombinant Slit2. Slit2 siRNA, srGAP1 siRNA, or scrambled sequence were administered by i.c.v. injection (20μL, 250 pmol/μL, 1 μL/min) 24 h prior to GMH at the following coordinates relative to bregma: 1.0 mm (rostral), 1.0 mm (left lateral), and 1.8 mm (in depth).

### Preparation of brain tissues

At 5 days after GMH induction, deeply anesthetized (5% isoflurane) animals were subjected to transcardial perfusion with ice-cold PBS for Western blotting or with ice-cold PBS followed by 10% formalin for immunohistochemical assays. The forebrain was rapidly frozen in liquid nitrogen, preserved at − 80 °C (for Western blotting) or fixed at 10% formalin for 3 days followed by dehydration with sucrose solution (30%) at 4 °C for 3 days (for immunofluorescence). The forebrain sample for immunofluorescence was embedded in the optimal cutting temperature compound and preserved at − 20 °C.

### Protein extraction and Western blotting

Forebrain samples were dissolved within RIPA lysis buffer (Santa Cruz Biotechnology, Dallas, TX, USA), and supernatants from the homogenates were collected after centrifugation at 15,000 RPM at 4 °C for 30 min. Protein concentrations were determined using a detergent compatibility assay kit (Bio-Rad, Irvine, CA, USA). Thirty micrograms of protein per sample were loaded into wells of 4–20% gels, run for 30 min at 80 V and then 60 min at 120 V, followed by protein transfer onto 0.45 mm nitrocellulose membranes for 60–120 min (Bio-Rad, Hercules, CA, USA). The membranes were incubated for 2 h in 5% nonfat milk in Tris-buffered saline containing 0.1% Tween 20. The following primary antibodies were incubated overnight at 4 °C: anti-Slit2 (cat: ab7665, 1:1000), anti-myeloperoxidase (MPO) (cat: ab65871, 1:1000), anti-IL-6 (cat: ab9324, 1:1000), anti-TNF-α (cat: ab6671,1:1000), anti-Cleaved Caspase-3(cat: ab2302, 1:500) (all from Abcam, Cambridge, MA, USA), anti-Robo1(cat: sc-25672, 1:200), anti-Macrophage/Monocytes (cat: sc-59103, 1:200), and anti-ICAM1(cat: sc-8439, 1:200) (both from Santa Cruz Biotechnology, Dallas, TX, USA). Anti-β-actin antibodies (cat: sc-69879, 1:1000; Santa Cruz Biotechnology, Dallas, TX, USA) were used as loading control. Membranes with proper secondary antibodies (cat: sc-2357/ sc-525409, 1:4000, Santa Cruz Biotechnology, Dallas, TX, USA) were incubated for 1 h at room temperature. Then enhanced chemiluminescent solution (GE Healthcare and Life Science, Piscataway, NJ, USA) was applied to membranes. Protein exposed to X-ray photographic films were analyzed by ImageJ software (4.0, Media Cybernetics, Silver Spring, MD, USA) for the relative density.

### Immunofluorescent staining

Following PBS perfusion and post-fixation of the forebrain samples in formalin, 10-µm-thick coronal sections were cut using a cryostat (Leica Microsystems LM3050S, Wetzlar, Germany). Immunofluorescence staining was performed as previously described [24]. Briefly, the sections were incubated overnight at 4 °C with the following primary antibodies: anti-Slit2 (cat: ab7665, 1:100), anti-neuronal nuclei (NeuN, cat: ab104224, 1:200, neuron marker), anti-glial fibrillary acidic protein (GFAP, cat:ab279290, 1:200, astrocyte marker), anti-myeloperoxidase (MPO, cat: ab65871, 1:200, neutrophil marker) (all from Abcam, Cambridge, MA, USA), anti-Robo1 (cat:sc-25672, 1:100), and anti- differentiation 68 (cat: sc-59103, CD-68, 1:100 macrophages/monocytes marker) (all from Santa Cruz Biotechnology, Dallas, TX, USA). The sections were then incubated with fluorescein isothiocyanate (FITC)-(cat:715-095-151/115-095-003) and Alexa Fluor® 594-conjugated secondary antibodies (cat:115-585-003/711-587-003,1:100; Jackson ImmunoResearch, West Grove, PA, USA) for 1 h at room temperature. TUNEL staining was performed using TUNEL assay kit based on manufacture instruction (cat: C10617; ThermoFisher Scientific, Waltham, MA). All brain slides were visualized using a fluorescence microscope (Olympus BX51, Japan).

For quantitative analysis of immunofluorescent staining, MPO-positive cells within peri-lesion region were counted and averaged from randomly selected three fields of view/brain slice, three brain slices/rat.

### Brain water content measurement

Brain edema was evaluated using the wet weight/dry weight method at 24 h after GMH [32]. Briefly, after the animals were killed, the brain was removed immediately, and a coronal tissue slice (4 mm thick) around the injection needle tract was cut. Each section was dissected into three parts: right hemisphere, left hemisphere, and cerebellum. The brain tissue weights were determined as soon as they were removed and then dried at 100 °C for 24 h to determine the dry weight. Finally, the brain water content (%) in each region was calculated using the following formula: [(wet weight − dry weight)/wet weight] × 100.

### Cdc42 activity assay

A pull-down assay was performed using a Cdc42 activity assay kit (cat: STA-402, Cell Biolabs, San Diego, CA, USA) as previously described [33]. Briefly, samples were mixed with p21-activated protein kinase (PAK1)-p21-binding domain (PBD) agarose beads and incubated at 4 °C for 1 h. The beads were resuspended in sample buffer, separated by 10% polyacrylamide gel electrophoresis, and transferred to a nitrocellulose membrane. The membrane was probed with the anti-Cdc42-specific antibody provided in the kit to detect the GTP-bound Cdc42.

### Neurobehavioral assessments

#### Short-term composite Neuroscore

The composite Neuroscore consisted of a sensorimotor value represented by the combined averages from negative geotaxis and righting reflex tests, as previously described [34, 35]. The values are expressed as a percentage of the sham group. The acquisition of developmental milestones was blindly assessed over 3 days. For negative-geotaxis, the time needed for complete rotation (180°) after placing the head down on a slope (20° angle) was recorded [36]. For the righting reflex, the time required for rat pups to completely roll over all four limbs after being placed on their backs was measured [36]. The allotted time was 60 s per trial (two trials/day) for these tests.

#### Long-term water maze and rotarod tests

Morris water maze and rotarod tests were performed in a blinded manner to evaluate cognitive and sensorimotor deficits in rats between 21 and 28 days after GMH induction as previously described [37].

Simply put, the rats were placed in a circular pool filled with water (110 cm in diameter) and trained to find a visible platform (11 cm in diameter) after these cue trials, the platform was slightly submerged and the rats were allowed to find the location of the platform in 10 trials per day for 4 consecutive days. The platform was removed from the pool at the end (probe trial). An overhead camera with a computerized tracking system (Noldus Ethovision, Noldus, Tacoma, WA, USA) recorded the swimming path and measured the swimming distance and time spent within the probe quadrant.

For Rotarod Test, briefly, rats were placed on a rotarod (Columbus Instruments, Columbus, OH, USA) consisting of a rotating horizontal cylinder (7 cm in diameter). The rats were required to move along the rotating cylinder to avoid falling. Rotarod testing started at either 5 or 10 RPM with an acceleration of 2 RPM every 5 s. A photobeam circuit detected the fall, and the latency of this event was recorded for each animal.

### Statistical analysis

Statistical analysis was performed using Sigma Plot 10.0 and Sigma Stat 3.5 (Systat Software, San Jose, CA, USA). Data are presented as mean ± SEM and were evaluated using a one-way ANOVA followed by Tukey’s or Student–Newman–Keuls tests. *P* values less than 0.05 were considered statistically significant.

## Results

All sham-operated rats survived. One GMH rat died of oxygen deficit. Overall mortality in the total study was 0.64%.

### Increased expressions of endogenous Slit2, Robo1 and srGAP1 in brain following GMH

**Fig. 1:**
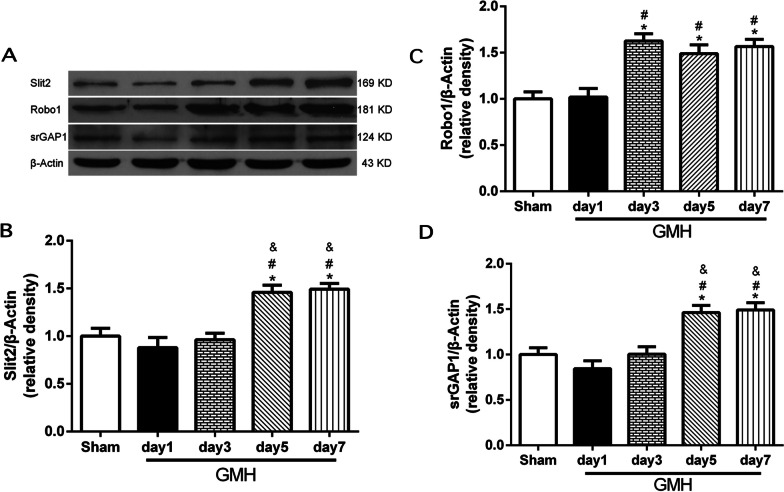
Expression of endogenous Slit2, Robo1 and srGAP1 in the brain after GMH. Representative Western blotting images (**A**) and quantitative analysis showed that an increased Slit2 expression at day 5 and day 7 after GMH (**B**), an increased Robo1 expression at day 3, day 5 and day 7 after GMH (**C**), and an increased srGAP1 expression at day 5 and day 7 after GMH (**D**). *N* = 6/group. Mean ± SEM. ANOVA, Tukey. *P < 0.05 vs. Sham, ^#^*P* < 0.05 vs. GMH day 1, ^&^*P* < 0.05 vs. GMH day 3

A time-course study was conducted to determine the brain expressions of Slit2, Robo1 and srGAP1 on days 1, 3, 5, and 7 following GMH. Endogenous brain protein expression levels of Slit2 and srGAP1 were increased on day 5 and persisted on day 7 following GMH (*P* < 0.05 sham vs. GMH; Fig. 1A, B, D). Similarly, endogenous brain Robo1 protein expression level was significantly increased and peaked on day 3 after GMH (*P* < 0.05 sham vs. GMH; Fig. 1A, C).

### Cell co-localization of endogenous Slit2 and Robo1 in brain following GMH

**Fig. 2:**
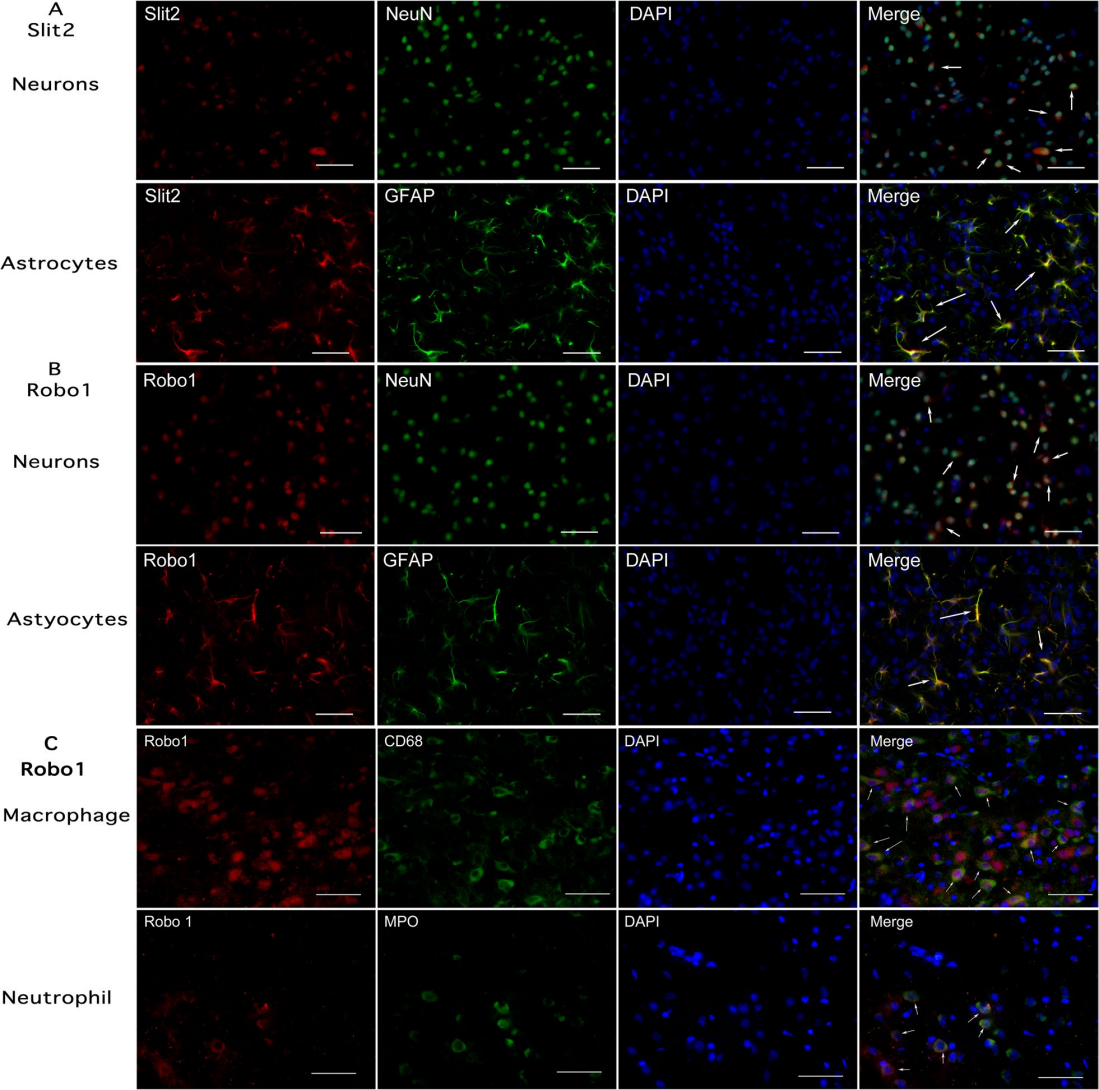
Cellular locations of Slit2 and Robo1 in brain at 5 days after GMH. Representative microphotograph of immunofluorescence staining showed a co-localization of Slit2 (**A**) and Robo1 (**B**) with neuron (NeuN) or astrocyte (GFAP), respectively. Scale bar = 100 μm. Robo1 (**C**) was colocalized with infiltrating CD68-positive macrophages or MPO positive neutrophil, respectively. Scale bar = 200 μm. Arrows indicate merged cells

Double-immunofluorescence staining showed that Slit2 was expressed on neurons and astrocytes (Fig. 2A) on day 5 after GMH. Robo1 was also expressed on both neurons and astrocytes (Fig. 2B). Furthermore, Robo1 colocalized with MPO positive neutrophils and CD68-positive macrophages in the brain tissues (Fig. 2C). Low magnification microphotographs showed relatively even distribution characteristics of Robo1-positive staining at peri-lesion and areas remote to lesion (Additional file 1: Fig. S1).

### Knockdown of endogenous brain Slit2 worsened brain edema and short-term neurobehavioral deficits following GMH

**Fig. 3:**
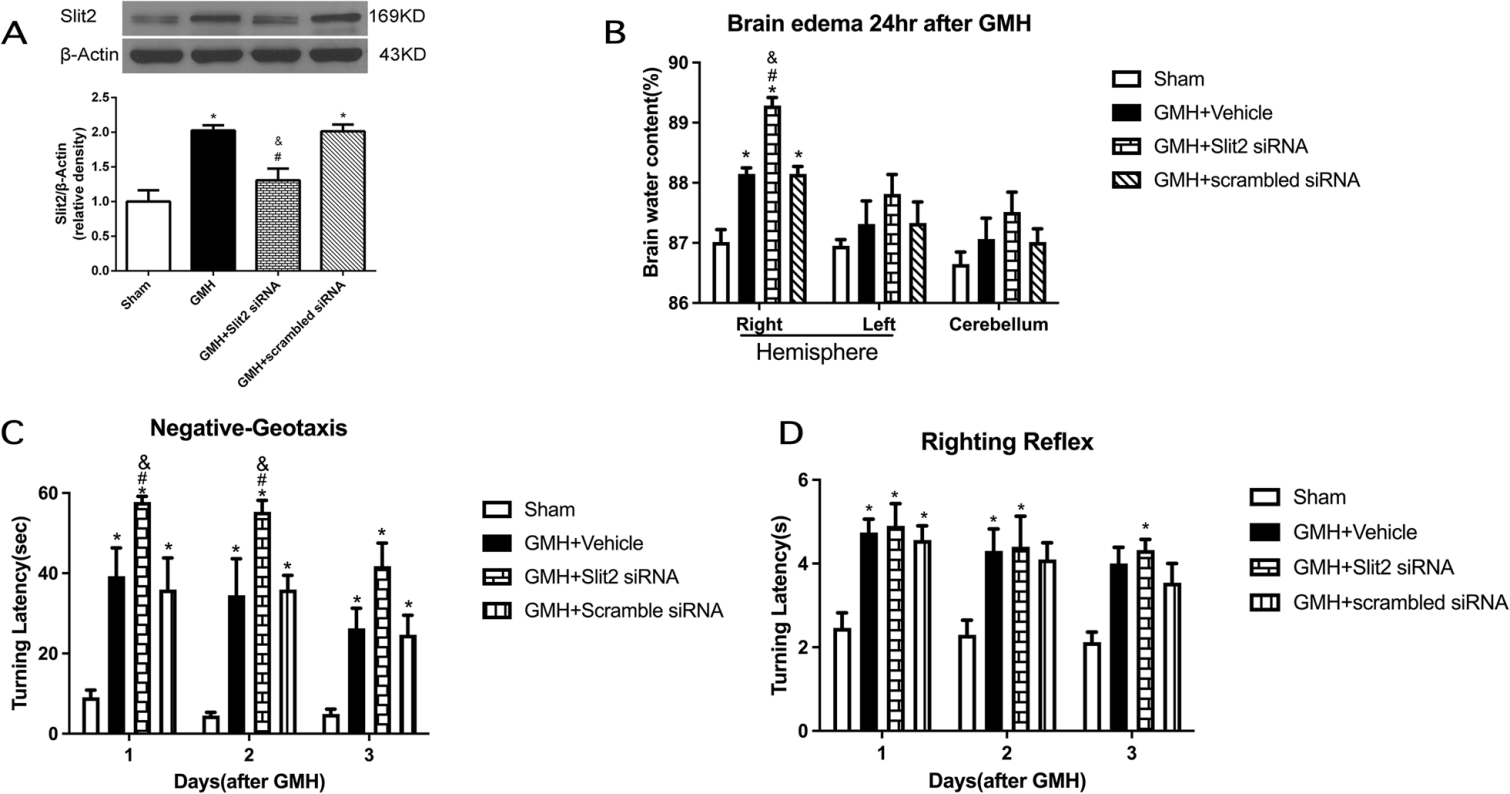
Effects of endogenous brain Slit2 knockdown in GMH. **A** Representative Western blotting image and quantitative analysis showed that Slit2 siRNA significantly reduced the brain Slit2 expression. Endogenous brain Slit2 knockdown worsened brain edema at 24 h after GMH (**B**) and aggravated neurobehavioral performance in the negative-geotaxis test (**C**) at 1 day and 2 days after GMH. **D** There was not significant different among all GMH groups in righting-reflex test during the 3 days after GMH. *N* = 6/group. Mean ± SEM. ANOVA, Tukey. *P < 0.05 vs. Sham, ^#^*P* < 0.05 vs. GMH + Vehicle, ^&^*P* < 0.05 vs. GMH + scrambled siRNA

Slit2 knockdown by Slit2 siRNA effectively suppressed the brain expression of Slit2 (*P* < 0.05 GMH + Slit2 siRNA vs. GMH + scramble siRNA, GMH; Fig. 3A). Brain water content of ipsilateral (right) hemisphere was significantly increased at 24 h after GMH (*P* < 0.05 sham vs. GMH; Fig. 3B). GMH rats that received Slit2 siRNA had significantly higher brain water content (*P* < 0.05 GMH + Slit2 siRNA vs. GMH + scramble siRNA,  GMH + Vehicle; Fig. 3B). The negative-geotaxis test showed worse neurological function in the GMH + Slit2 siRNA group than that in the GMH + scrambled siRNA group at 1 and 2 day after GMH (*P* < 0.05; Fig. 3C). However, there were no significant differences among the groups in the righting reflex test during 3 days after GMH (Fig. 3D).

### Recombinant Slit2 administration improved short-term neurological deficits following GMH

**Fig. 4:**
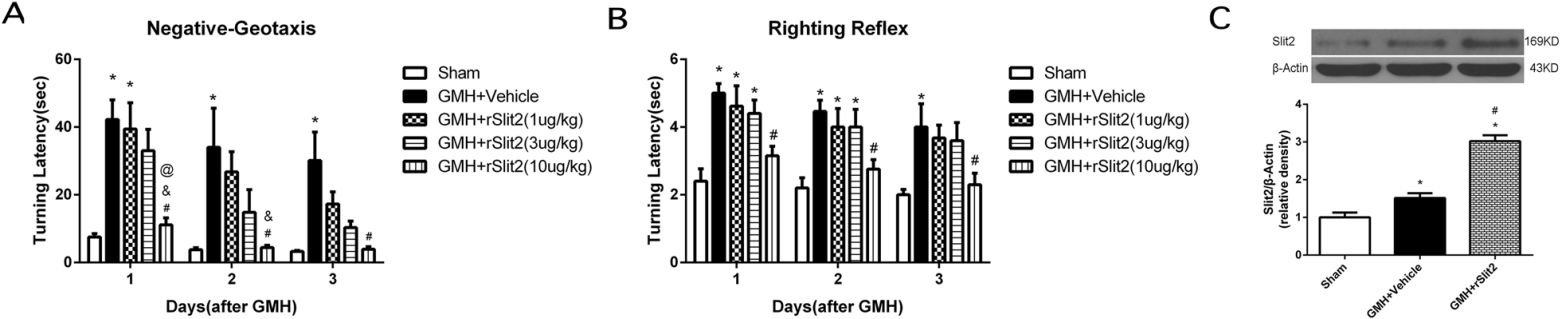
Effects of recombinant Slit2 on neurobehavior after GMH. Recombinant Slit2 (10 μg/kg) improved the performance in the negative-geotaxis (**A**) and righting-reflex (**B**) at 1, 2 and 3 days after GMH. N = 6/group. *P < 0.05 compared to Sham, #*P* < 0.05 compared to GMH + Vehicle, rSlit2 (1 μg/kg), rSlit2 (3 μg/kg). **C** Brain Slit2 expression was increased in the rSlit2 treatment group, indicating that rSlit2 (10 μg/kg) was successfully delivered into the brain by intranasal administration. *N* = 4/group. Mean ± SEM. ANOVA, Tukey. *P < 0.05 vs. Sham, #*P* < 0.05 vs. GMH + Vehicle

Short-term neurological function evaluated by the negative-geotaxis (Fig. 4A) and righting reflex (Fig. 4B) tests worsened on days 1, 2 and 3 after GMH (*P* < 0.05 sham vs. GMH + Vehicle). Although all doses of rSlit2 improved negative-geotaxis and righting reflex performances from day 1 to 3 after GMH, only rSlit2 10 μg/kg showed a significant result at day 1 and 2 (*P* < 0.05 GMH + Vehicle vs. GMH + Slit2). In addition, the delivery efficiency of intranasal administration of rSlit2 (10 μg/kg) was validated. After intranasal administration of rSlit2, Slit2 expression was significantly higher in the rSlit2 treated GMH group than that in the vehicle treated GMH group on day 5 (*P* < 0.05; Fig. 4C), indicating that intranasally administered rSlit2 (10 μg/kg) was successfully delivered into the brain.

### Recombinant Slit2 reduced neuronal death following GMH

**Fig. 5:**
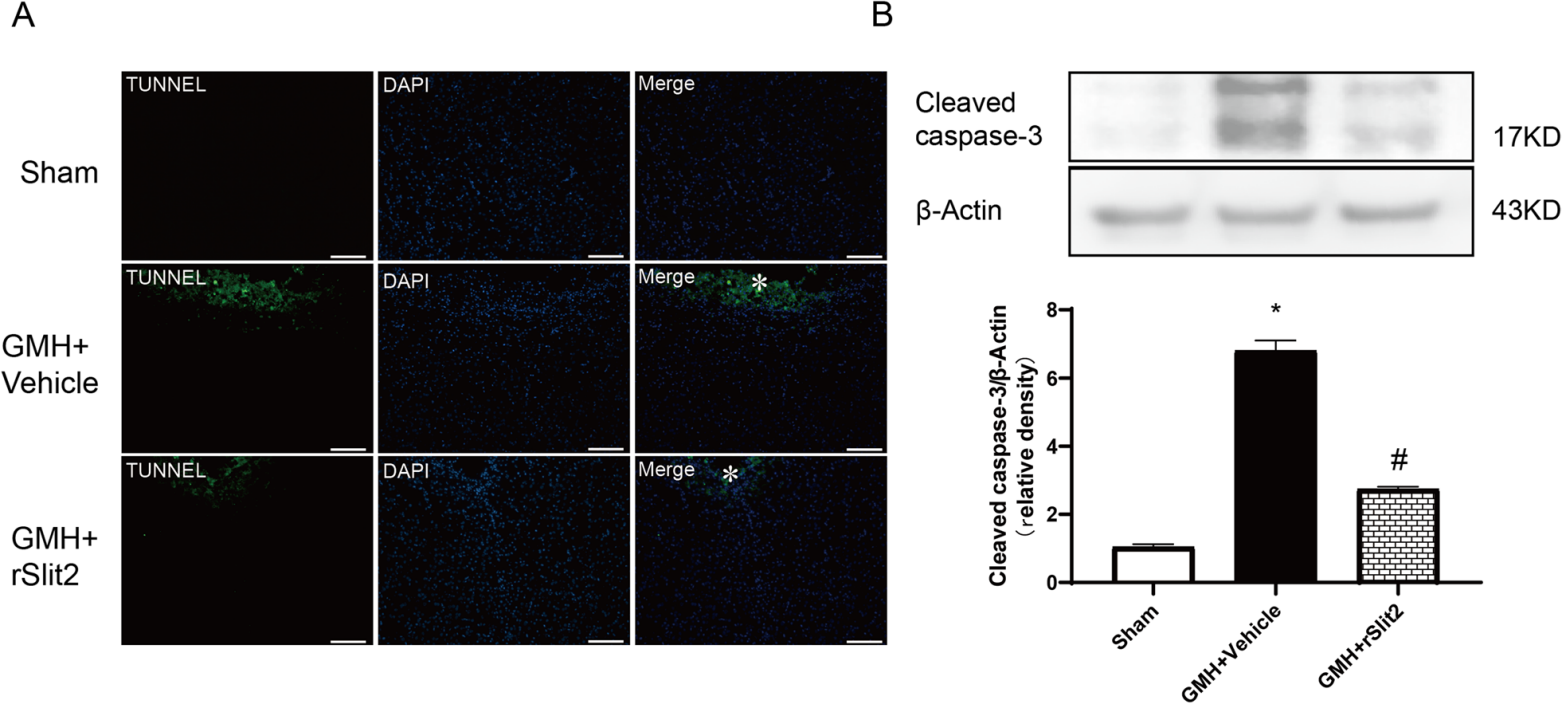
Effects of recombinant Slit2 on neuronal death in brain at 5 days after GMH. TUNEL staining showed the degree of DNA breakage (**A**) was aggravated after GMH, which were reduced by rSlit2. “*” symbol indicates the lesion location on the representative microphotograph of brain slice. Scale bar = 100 μm. Western blotting image and quantitative analysis showed that brain cleaved caspase-3 (**B**) expressions increased after GMH, which were reduced by rSlit2. *N* = 6/group. Mean ± SEM. ANOVA, Tukey. *P < 0.05 vs. Sham, #*P* < 0.05 vs. GMH + Vehicle

There were TUNEL-positive staining at 5 days after GMH, reduced in rSlit2 10 μg/kg-treated GMH rats (Fig. 5A). Similarly, in the brain tissue of GMH rats, there was a significant increase in cleaved caspase-3 protein level (*P* < 0.05 sham vs. GMH; Fig. 5B), while rSlit2 decreased it (*P* < 0.05 GMH + Vehicle vs. GMH+rSlit2; Fig. 5B). rSlit2 treatment effects on TUNEL-positive staining and cleaved caspase-3 protein level in the brain tissues were also observed at 1 day after GMH (Additional file 1: Fig. S2).

### Recombinant Slit2 reduced brain expressions of proinflammatory cytokines/chemokines and neutrophil infiltrations following GMH

**Fig. 6:**
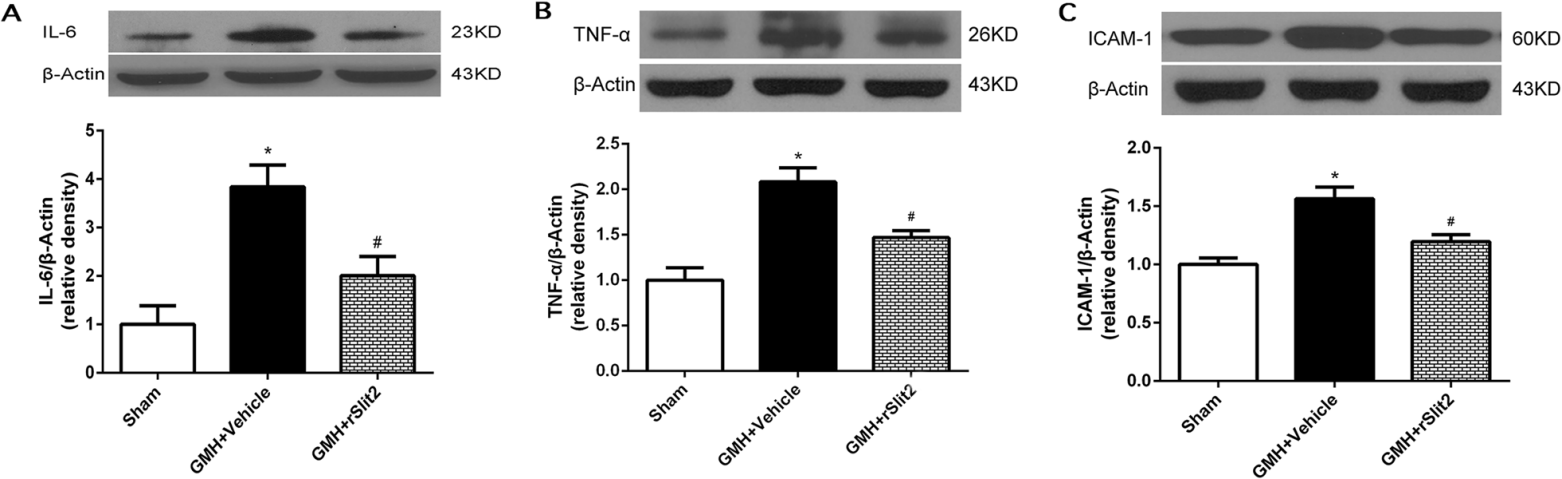
Effects of recombinant Slit2 on proinflammatory cytokines in brain at 5 days after GMH. Western blotting image and quantitative analysis showed an increase in brain IL-6 (**A**), TNF-α (**B**) and ICAM-1 (**C**) expressions after GMH, which were reduced by recombinant Slit2 treatment. *N* = 6/group. Mean ± SEM. ANOVA, Tukey. *P < 0.05 vs. Sham, #*P* < 0.05 vs. GMH + Vehicle

**Fig. 7:**
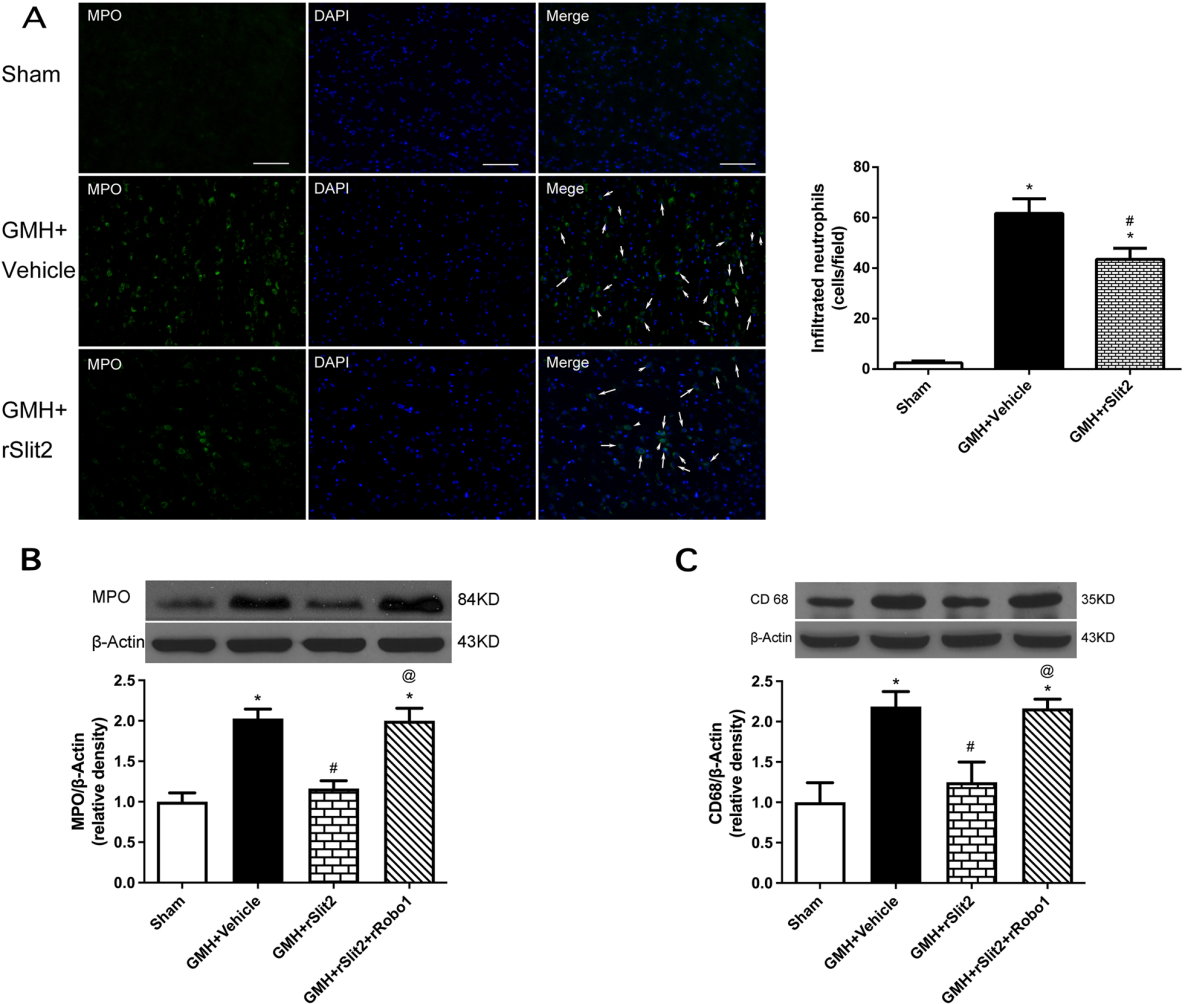
Effects of recombinant Slit2 on brain peripheral immune cells infiltration at 5 days after GMH. **A** Representative microphotograph and quantitative analysis of immunofluorescence staining showed that fewer MPO positive neutrophils were visualized in the brain tissue of GMH + rSlit2 group compared to GMH + Vehicle group. Scale bar = 100 μm; Western blotting analysis showed that recombinant Slit2 down-regulated brain neutrophil marker MPO (**B**) and macrophage marker CD68 (**C**) after GMH and such effects were reversed by rRobo1 co-administration. *N* = 6/group. Mean ± SEM. ANOVA, Tukey. *P < 0.05 vs. Sham, ^#^*P* < 0.05 vs. GMH + Vehicle, ^@^*P* < 0.05 vs, GMH + rSlit2

The expressions of proinflammatory cytokines/chemokines TNF-α, IL-6, and ICAM-1 in the brain was quantified using Western blotting at 5 days after GMH. Protein expression levels were significantly higher after GMH (*P* < 0.05 sham vs. GMH + Vehicle; Fig. 6A–C). Recombinant Slit2 10 μg/kg significantly reduced the upregulations of brain TNF-α, IL-6, and ICAM-1 after GMH (*P* < 0.05 GMH + Vehicle vs. GMH + rSlit2). Immunofluorescence staining showed greater MPO-positive cells in brains of GMH rats than shams, but there were fewer cells positively stained for MPO in the brain of rSlit2 (10 μg/kg)-treated GMH animals than that of the vehicle-treated GMH animals at 1 day (Additional file 1: Fig. S3A) and at 5 day (Fig. 7A and Additional file 1: Fig. S3B) after GMH.

### Beneficial effects of recombinant Slit2 following GMH were reversed by recombinant Robo1 co-administration or srGAP1 siRNA

**Fig. 8:**
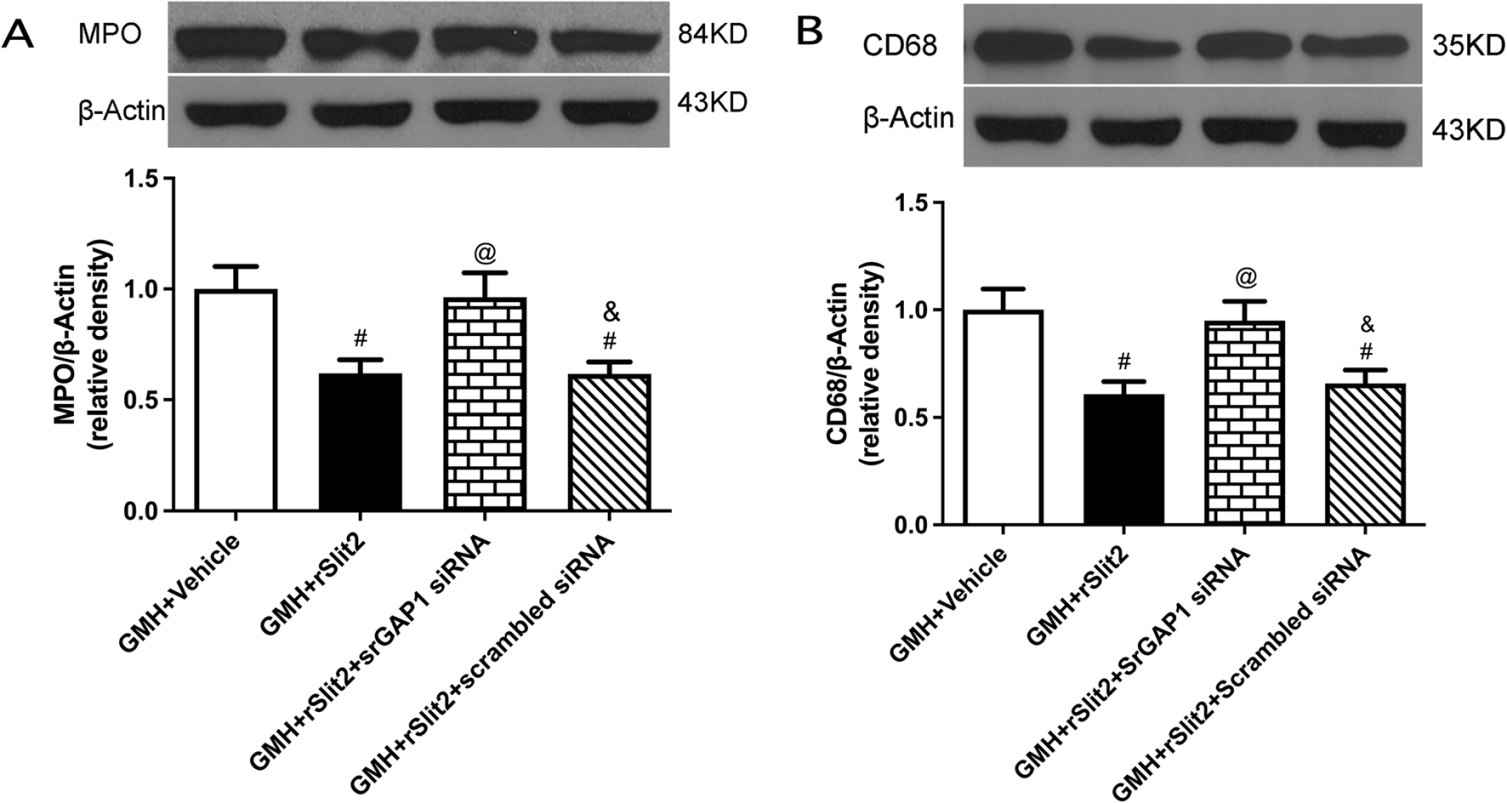
Effects of srGAP siRNA on recombinant Slit2-mediated downregulation of peripheral immune cells infiltration into brain. Western blotting analysis showed that the downregulation of brain MPO (**A**) and CD68 (**B**) expressions by recombinant Slit2 were reversed by srGAP1 siRNA in GMH rats. N = 6/group. Mean ± SEM. ANOVA, Tukey. ^#^*P* < 0.05 vs. GMH + Vehicle, ^@^*P* < 0.05 vs. GMH + rSlit2. ^&^*P* < 0.05 vs. GMH + rSlit2 + srGAP1 siRNA

We investigated the role of Robo1–srGAP1 pathway in rSlit2-mediated anti-inflammation after GMH. The expression of neutrophil marker MPO (Fig. 7A, B), and macrophage marker CD68 (Fig. 7C) were significantly increased after GMH (*P* < 0.05 sham vs. GMH + Vehicle; Fig. 7). rSlit2 (10 μg/kg) significantly reduced the expression of both markers (*P* < 0.05 GMH + Vehicle vs. GMH + rSlit2; Fig. 7). Either recombinant Robo1 co-administration or srGAP1 siRNA abolished the effects of recombinant Slit2 with higher expressions of MPO and CD68 (*P* < 0.05 GMH + Slit2 vs. GMH + Robo1 + rSlit2, Fig. 7; *P* < 0.05 GMH + rSlit2 vs. GMH + srGAP1 siRNA + rSlit2, Fig. 8). The lower magnification microphotograph showed that neutrophils are concentrated in peri-lesion area (Figure S3). Similar to Day 5, MPO-positive cells were evident in the peri-lesion area of GMH rats on day 1, which was reduced by rSlit2 treatment (Additional file 1: Fig. S3).

**Fig. 9:**
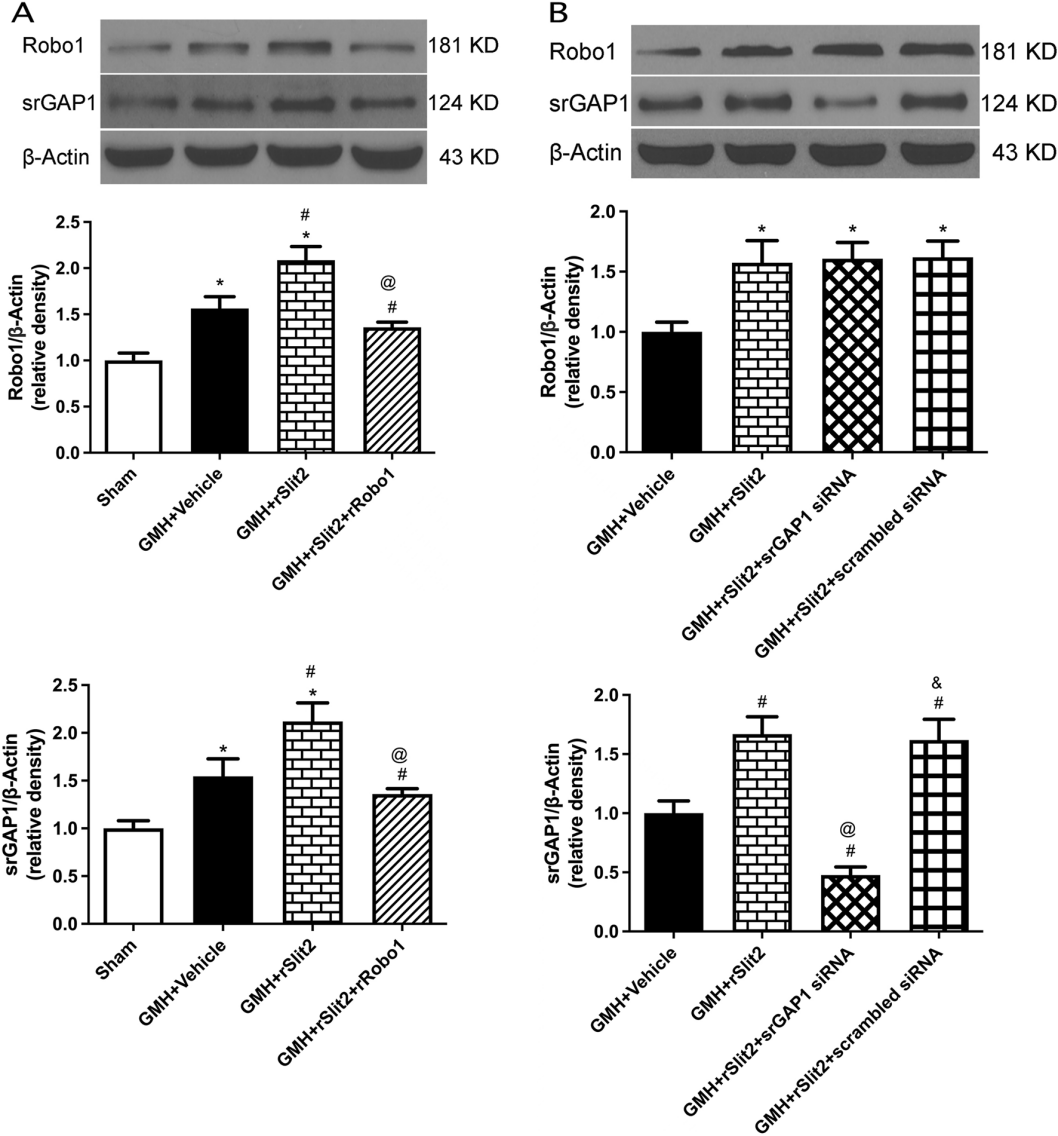
Western blotting analysis of brain Robo1 and srGAP1 expressions at 5 days after GMH. **A** Brain expressions of Robo1 and srGAP1 were significantly increased after GMH compared to Sham, which were further elevated in GMH + rSlit2 group. Recombinant Robo1 co-administration with rSlit2 reversed the upregulation effects of rSlit2 of on Robo1 and srGAP1. **B** Brain srGAP1 expression was increased in GMH + rSlit2 and GMH + Slit2 + scrambled siRNA groups compared to GMH. The administration of srGAP1 siRNA decreased the expression of srGAP1, but not Robo1 in brain after GMH. *N* = 6/group. Mean ± SEM. ANOVA, Tukey. *P < 0.05 vs. Sham, #*P* < 0.05 vs. GMH + Vehicle, ^@^*P* < 0.05 vs. GMH + rSlit2, ^&^*P* < 0.05 vs. GMH + rSlit2 + srGAP1 siRNA

Co-administration of recombinant Robo1 with recombinant Slit2 reversed the rSlit2 effects on both brain expressions of Robo1 and srGAP1 after GMH (*P* < 0.05 GMH + rSlit2 vs. GMH + rSlit2 + rRobo1; Fig. 9A). The administration of srGAP1 siRNA reversed rSlit2 effect on brain srGAP1expression, but had no effect on the brain protein level of Robo1 (*P* < 0.05 GMH + rSlit2 vs. GMH + rSlit2 + srGAP1 siRNA; Fig. 9B).

**Fig. 10:**
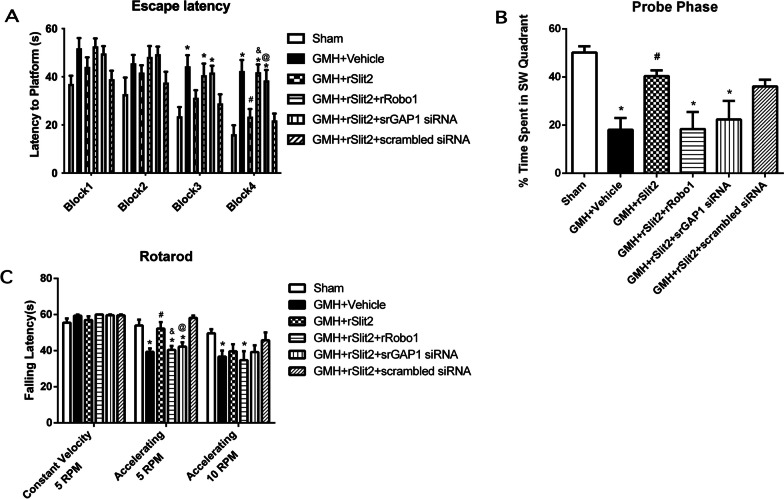
Effects of recombinant Slit2 on long-term neurobehaviors after GMH. Recombinant Slit2 improved the performances on Water Maze test (**A**, **B**) and Rotarod test (**C**) in GMH rats, which were reversed either by co-administration of rRobo1 or srGAP1 siRNA. *N* = 6/group. Mean ± SEM. ANOVA, Tukey. *P < 0.05 vs. Sham, ^#^*P* < 0.05 vs. GMH + Vehicle, ^&^*P* < 0.05 vs. GMH + rSlit2, ^@^*P* < 0.05 vs. GMH + rSlit2 + scrambled siRNA

Long-term neurobehavioral function was evaluated using the Morris water maze and rotarod tests on days 21–28 after GMH. Animals of GMH + Vehicle, GMH + rSlit2 + Robo1, and GMH + rSlit2 + srGAP1 siRNA groups took a longer period of time to find the platform than sham animals in the Morris water maze trials (*P* < 0.05; Fig. 10A). Furthermore, animals in the GMH + Vehicle group spent less time in the target quadrant during the probe trials than the animals in the sham group (*P* < 0.05 sham vs. GMH + Vehicle; Fig. 10B). Animals received rSlit2 treatment spent more time in the target quadrant during the probe trials than vehicle-treated animals (*P* < 0.05). However, there were no significant differences in other groups. Similarly, animals in groups of GMH + Vehicle, GMH + rSlit2 + rRobo1, and GMH + rSlit2 + srGAP1 siRNA fell off the rotating cylinder earlier than the sham animals at a starting speed of 5 RPM (*P* < 0.05; Fig. 10C). Animals receiving recombinant Slit2 treatment performed better on the rotarod test than the animals in the vehicle group (*P* < 0.05).

### The inhibition effects of recombinant Slit2 on Cdc42 activity following GMH were reversed by recombinant Robo1 co-administration or srGAP1 siRNA

**Fig. 11:**
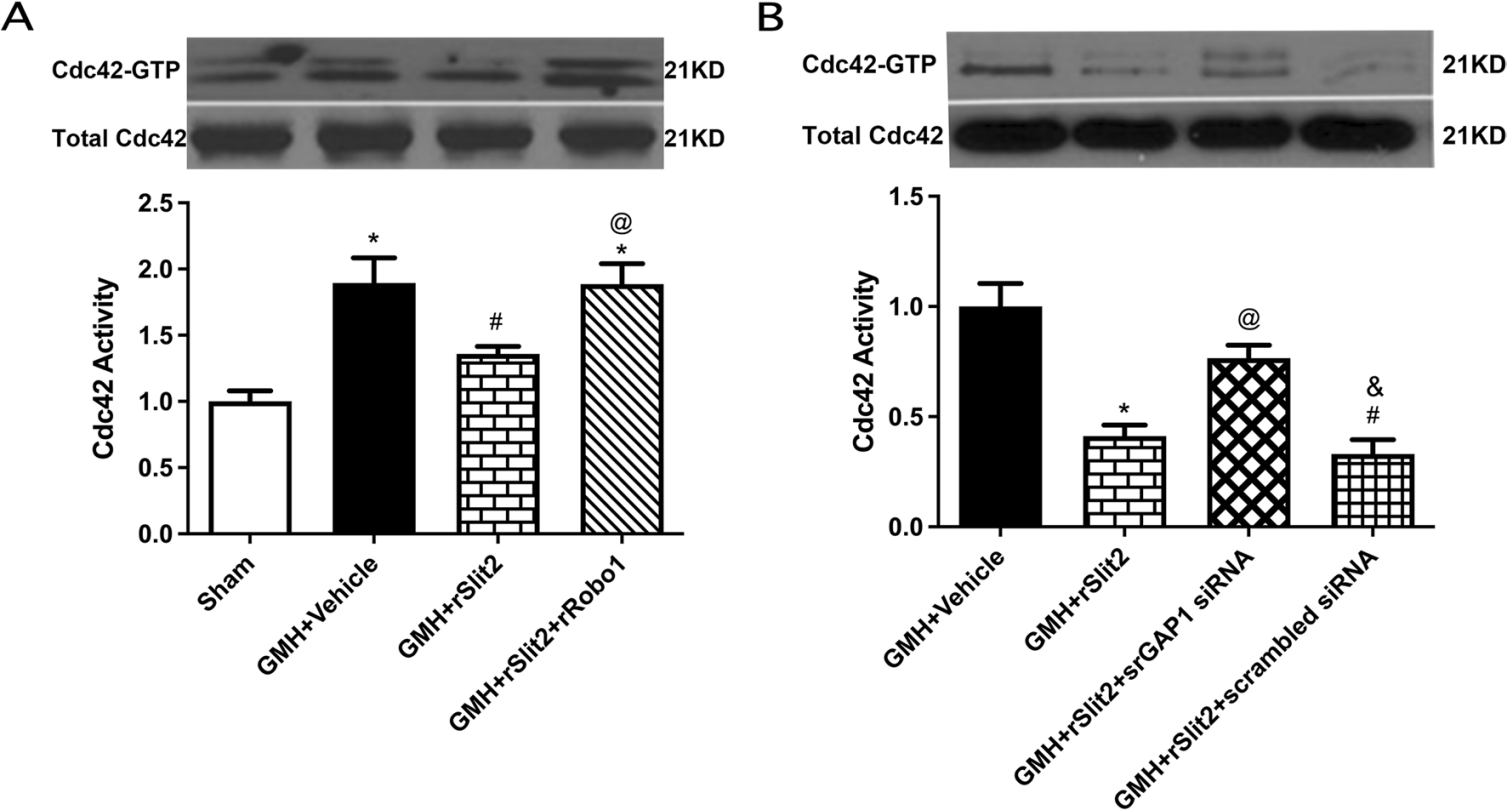
Brain Cdc42 activity assessment at 5 days after GMH. **A** There was an elevation of Cdc42 activity in brain after GMH, which reduced by recombinant Slit2 treatment. Recombinant Robo1 co-administration blocked the inhibition effect of recombinant Slit2 on brain Cdc42 activity. **B** srGAP1 siRNA, but not scrambled siRNA, blocked the inhibition effect of recombinant Slit2 on brain Cdc42 activity. *N* = 6/group. Mean ± SEM. ANOVA, Tukey. *P < 0.05 vs. Sham, ^#^*P* < 0.05 vs. GMH + Vehicle, ^@^*P* < 0.05 vs. GMH + rSlit2, ^&^*P* < 0.05 vs. GMH + rSlit2 + srGAP1 siRNA

Brain Cdc42 activity was significantly increased at 5 days after GMH (*P* < 0.05 sham vs. GMH + Vehicle+ GMH; Fig. 11A), which was reduced in rSlit2 10 μg/kg-treated GMH rats (*P* < 0.05 GMH+ Vehicle vs. GMH + rSlit2). Recombinant Robo1 (3 μg/kg) partially reversed the reduction in Cdc42 activity induced by rSlit2 (*P* < 0.05 GMH + Robo1 + rSlit2 vs. GMH + rSlit2 10 μg/kg; Fig. 11A). srGAP1 siRNA also reversed the reduction in brain Cdc42 activity induced by rSlit2 (*P* < 0.05 GMH + srGAP1 siRNA vs. GMH + rSlit2, GMH + rSlit2 +scramble siRNA; Fig. 11B).

## Discussion

In this study, we investigated the therapeutic potential of recombinant Slit2 protein in suppressing the neuroinflammatory response following GMH in rats. We observed that the brain expression of endogenous Slit2 was significantly increased after GMH. The knockdown of endogenous brain Slit2 exacerbated GMH-induced brain edema and short-term neurological deficits. Recombinant Slit2 treatment after GMH attenuated neuronal death, neuroinflammation and neurological deficits, but inhibited Cdc42 activity and reduced the cerebral infiltration of peripheral immune cells after GMH. The beneficial effects of rSlit2 treatment were reversed by co-administration with the decoy receptor rRobo1 or the siRNA knockdown of downstream effector srGAP1 in the brain. According to these results, brain Slit2 signaling might provide an endogenous protective effect after GMH, and rSlit2 may serve as promising therapeutics to attenuate neuroinflammation after GMH.

Slit2 is a chemorepulsive element that facilitates axonal guidance and neuronal migration in the central nervous system (CNS) during development [38, 39]. The inflammatory responses in several systemic conditions, such as renal, peritoneal, and lung inflammation, have been demonstrated to be inhibited by Slit2 in many preclinical studies [40–42]. However, its efficacy in neuroinflammation in the context of GMH is still unclear. This study showed that endogenous brain Slit2 was increased from day 5 and persisted at day 7 after GMH. This protein localized on neurons and astrocytes in GMH rats. This finding matched earlier research that Slit2 was found to express on neurons and astrocytes [43]. Robo1 is a protein with a single transmembrane domain [44] that serves as a Slit receptor for axon guidance [27] and neuronal migration [19]. Robo1 is also expressed on the leukocyte that mediate the anti-migratory effects of Slit2 [45]. Our results also showed that Robo1 expression was positively correlated with Slit2 expression following GMH. Previous studies indicated the expression of Robo1 on neutrophils [41], lymphocytes [46], and mononuclear macrophages [30]. Consistently, we found that Robo1 was colocalized with infiltrating MPO-positive neutrophils and CD68-positive marcrophages in brains of GMH rats.

Slit was shown to be an endogenous inhibitory factor for leukocytes chemotaxis recently [41]. To clarify the role of endogenous Slit2 after GMH, Slit2 siRNA was used to knockdown the endogenous Slit2 in brain in prior to GMH induction. Endogenous Slit2 knockdown worsened GMH-induced brain edema of the ipsilateral hemisphere and sensorimotor dysfunction in the negative-geotaxis test. The endogenous brain Slit2 seems to be elevated as a potential compensatory mechanism, although the protective effects were not sufficient to counteract brain injury after GMH. Firstly, Slit2 and its receptors including Robo1 and Robo4 are labeled to regulate the mobility and permeability of endothelial cells and other cell types. The brain secretory protein Slit2 can be secreted into the CSF and eventually into the bloodstream. It could directly bind to Robo1 receptors in peripheral immune cells, suppressing their migration to the brain parenchyma [26]. In addition, previous studies have shown that Slit2 can exert anti-inflammatory effects mediated by the dominant endothelial-specific receptor Robo4 [47]. Secondly, immune cells and proinflammatory factors can enter the brain parenchyma without any pathogen [48]. During viral infections or when certain autoimmune disorders develop in the CNS, activated T cells can cross the blood–brain barrier (BBB) and initiate both protective and unwanted inflammatory responses. When the BBB is damaged, many T cells and other inflammatory cells enter the CNS by encountering perivascular macrophages [48, 49]. Endogenous Slit2 may then be increased to compensate for endothelial barrier breakdown after GMH. By binding to the endothelial-specific receptor Robo4, Slit2 was proven to reduce endothelial barrier permeability [50, 51]. Thus, endogenous Slit2 knockdown worsened the outcomes in GMH rats.

The treatment effects of exogenous recombinant Slit2 were further evaluated after GMH. Three doses of recombinant Slit2 were examined by comparing the outcomes on day 1, day 2, and day 3 after GMH [44]. Recombinant Slit2 at dose of10 μg/kg improved the overall performance in the negative-geotaxis and righting reflex tests on both days 1 and 2 after GMH. In response to injury, leukocytes accumulate in the CNS [52, 53]. The activated microglia synthesize cytokines and inflammatory mediators that induce endothelial cells to upregulate ICAM-1 [54] and promote peripheral leukocyte adhesion to the brain and potentiates neuroinflammation [55]. Slit2 has been reported to inhibit leukocyte migration induced by chemokines [56, 57]. Our results demonstrated that recombinant Slit2 treatment reduced brain proinflammatory cytokines including TNF-α and IL-6, and ICAM-1, and the expressions of peripheral immune cell markers in brain tissues, such as MPO and CD68, in brain after GMH. Previous study showed recombinant Slit2 reduced neuronal death after experimental neonatal hypoxic-ischemic encephalopathy (HIE) [25]. Consistently, GMH induced neuron death and an increase in brain apoptotic marker at 1 and 5 days after GMH, which were attenuated by recombinant Slit2 treatment. Collectively, our finding suggests rSlit2 improved neurological outcomes by suppressing neuroinflammation and reducing neuronal apoptosis.

To better elucidate the anti-neuroinflammation mechanism of recombinant Slit2, the role of its single transmembrane domain receptor Robo1 [58] was investigated. A previous study showed that recombinant Robo1 acts as a decoy receptor by binding to recombinant Slit2, which resulted in less Slit2 available to bind to the Robo1 receptors on immune cells, thereby reducing its anti-migratory function [24]. In the present study, co-administering recombinant Robo1 with recombinant Slit2 as a decoy receptor neutralized the effects of recombinant Slit2 with increasing peripheral immune cells infiltration into the brain. This suggests that Slit2 regulates peripheral immune cell migration to the brain in a Robo1-dependent manner. Moreover, the downstream effector srGAP1 mediates the inhibitory effects of the Slit2-Robo1 interaction on migration [26]. Slit2 enhances the interaction of the SH3 domain in srGAP1 with the CC3 motif in Robo1, and this localization may induce inactivation of a small GTPase protein Cdc42, a member of the Rho-GTPase family, by converting the active GTP-bound form of Cdc42 to the inactive GDP-bound form of Cdc42 [59]. Cdc42 has been reported to function as a key mediator of cell migration [33]. The interaction between srGAP1 and the GTP-bound activated form of Cdc42 abolishes the directional mobility of the cell toward chemoattractant [17]. Consistently, our data revealed that Cdc42 activity was markedly inhibited by recombinant Slit2. When recombinant Robo1 was co-administered or srGAP1 was knocked down by siRNA, the effects of recombinant Slit2 on the inhibition of Cdc42 activity and peripheral immune cells migration/cerebral infiltration was reversed after GMH. This suggests that recombinant Slit2 may exert its anti-chemotactic and neuroprotective effects by activating the Robo1/ srGAP1 pathway to inhibit Cdc42 activity, which subsequently reduces peripheral immune cells infiltration into the brain after GMH. The activation Slit2/ Robo1/ srGAP1 signaling pathway was also shown to be the anti-apoptosis mechanism in the setting of HIE [25]. Therefore, we could not exclude such anti-neuronal apoptotic mechanism underlying the treatment effects of recombinant Slit2 after GMH, which needs future investigation.

In conclusion, our data showed that the endogenous brain chemorepulsive factor Slit2 was increased and served as a compensatory protective mechanism after GMH in rats. The recombinant Slit2 treatment reduced neuronal death and neuroinflammation. Specifically, the anti-neuroinflammation effect of recombinant Slit2 on suppressing Cdc42-mediated peripheral immune cell infiltration into brain was at least in part via the Robo1/ srGAP1 pathway. These results imply that recombinant Slit2 may have potentials as a therapeutic option for neonatal brain injuries.

### Supplementary Information

**Additional file 1:  Figure S1.** Cellular locations of Slit2 and Robo1 in brain at 5 days after GMH. Representative microphotograph of immunofluorescence staining showed a co-localization of Slit2 (**A**) or Robo1 (**B**) with neuron (NeuN) and astrocyte (GFAP) across the brain section, respectively. Co-localization of Robo1 (**B**) with CD68-positive macrophage or MPO positive neutrophil was presented at the peri-lesion brain area. “*” symbol indicates the location of lesion Scale bar=100 μm. **Figure S2.** Effects of recombinant Slit2 on neuronal death in brain at 1 days after GMH. TUNEL staining showed the aggravated degree of DNA breakage (**A**) after GMH, which were reduced by rSlit2 treatment. “*” symbol indicates the location of lesion. Scale bar=100μm. Western blotting image and quantitative analysis showed that brain cleaved caspase-3 (**B**) expressions increased after GMH, which were reduced by rSlit2 treatment. N=6/group. Mean±SEM. ANOVA, Tukey. *P<0.05 vs. Sham, #*P*<0.05 vs. GMH+Vehicle. **Figure S3.** Effects of recombinant Slit2 on peripheral immune cells infiltration into brain at 1 and 5 days after GMH. MPO immunofluorescence staining showed that MPO positive neutrophils were concentrated in the peri-lesion area at day 1(**A**) and day 5(B) after GMH, and  the number of MPO positive neutrophils were reduced by rSlit2 treatment. The lesion was located in the upper or/and left. Scale bar=100μm.

## Data Availability

The original datasets generated and/or analyzed during the current study are available from the corresponding author on reasonable request.
