# Supplementary material for: Recombinant Slit2 suppresses neuroinflammation and Cdc42-mediated brain infiltration of peripheral immune cells via Robo1–srGAP1 pathway in a rat model of germinal matrix hemorrhage
Source: J Neuroinflammation. 2023 Oct 29;20:249. doi: 10.1186/s12974-023-02935-2 (PMC10613398; doi:10.1186/s12974-023-02935-2)
Supplement: Supplementary file 1 — Additional file 1: Figure S1. Cellular locations of Slit2 and Robo1 in brain at 5 days after GMH. Representative microphotograph of immunofluorescence staining showed a co-localization of Slit2 (A) or Robo1 (B) with neuron (NeuN) and astrocyte (GFAP) across the brain section, respectively. Co-localization of Robo1 (B) with CD68-positive macrophage or MPO positive neutrophil was presented at the peri-lesion brain area. “*” symbol indicates the location of lesion Scale bar=100 μm. Figure S2. Effects of recombinant Slit2 on neuronal death in brain at 1 days after GMH. TUNEL staining showed the aggravated degree of DNA breakage (A) after GMH, which were reduced by rSlit2 treatment. “*” symbol indicates the location of lesion. Scale bar=100μm. Western blotting image and quantitative analysis showed that brain cleaved caspase-3 (B) expressions increased after GMH, which were reduced by rSlit2 treatment. N=6/group. Mean±SEM. ANOVA, Tukey. *P<0.05 vs. Sham, #P<0.05 vs. GMH+Vehicle. Figure S3. Effects of recombinant Slit2 on peripheral immune cells infiltration into brain at 1 and 5 days after GMH. MPO immunofluorescence staining showed that MPO positive neutrophils were concentrated in the peri-lesion area at day 1(A) and day 5(B) after GMH, and the number of MPO positive neutrophils were reduced by rSlit2 treatment. The lesion was located in the upper or/and left. Scale bar=100μm. [file 12974_2023_2935_MOESM1_ESM.docx]

Supplementary Figures and Legends


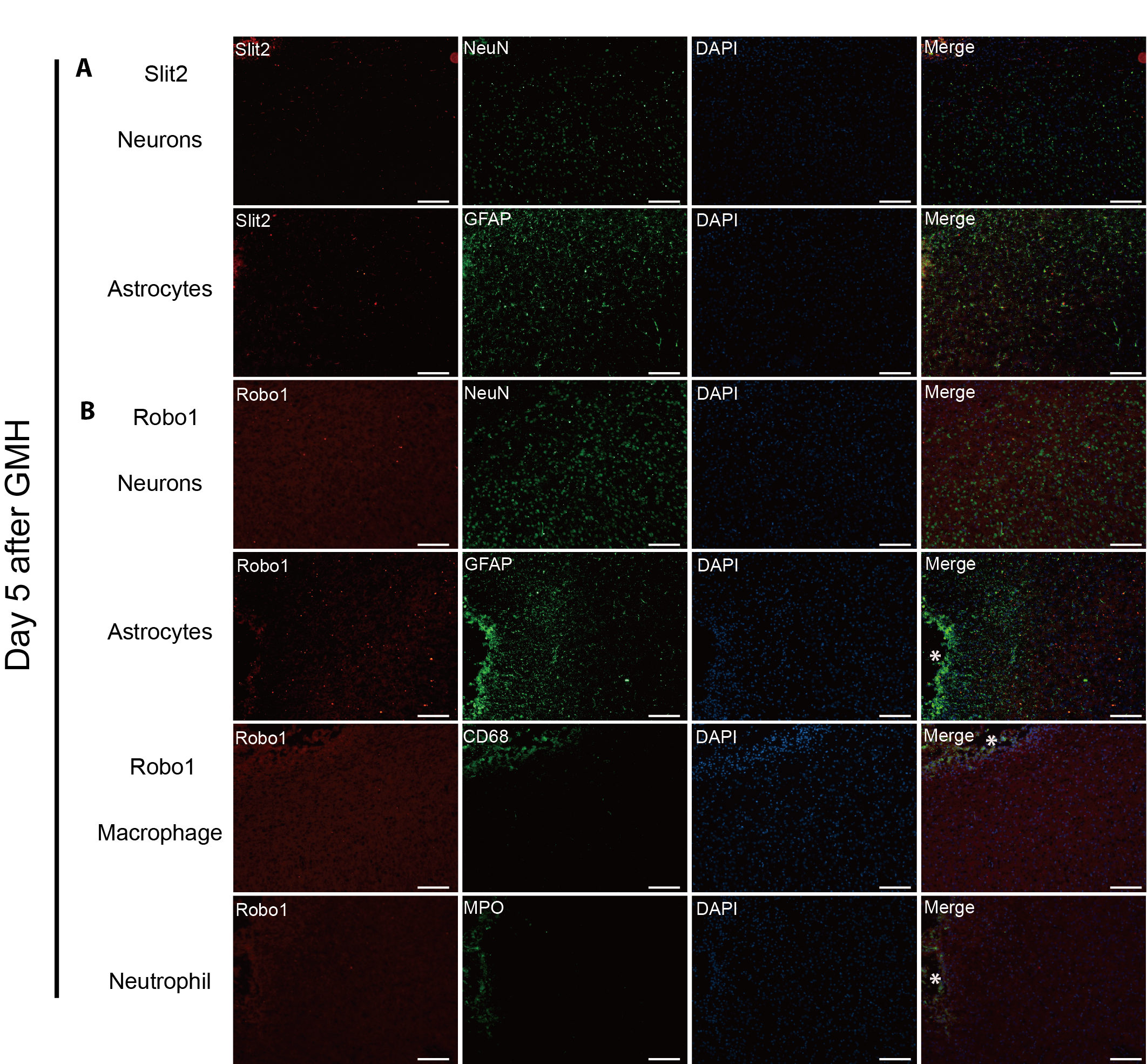


**Figure S1. Cellular locations of Slit2 and Robo1 in brain at 5 days after GMH.** Representative microphotograph of immunofluorescence staining showed a co-localization of Slit2 (A) with and Robo1 (B) with neuron (NeuN) and astrocyte (GFAP), respectively across the brain section. Co-localization of Robo1 (B) with CD68 positive macrophage or MPO positive neutrophil presented at the peri-lesion brain area. “*” symbol indicates the location of lesion Scale bar=100 μm.


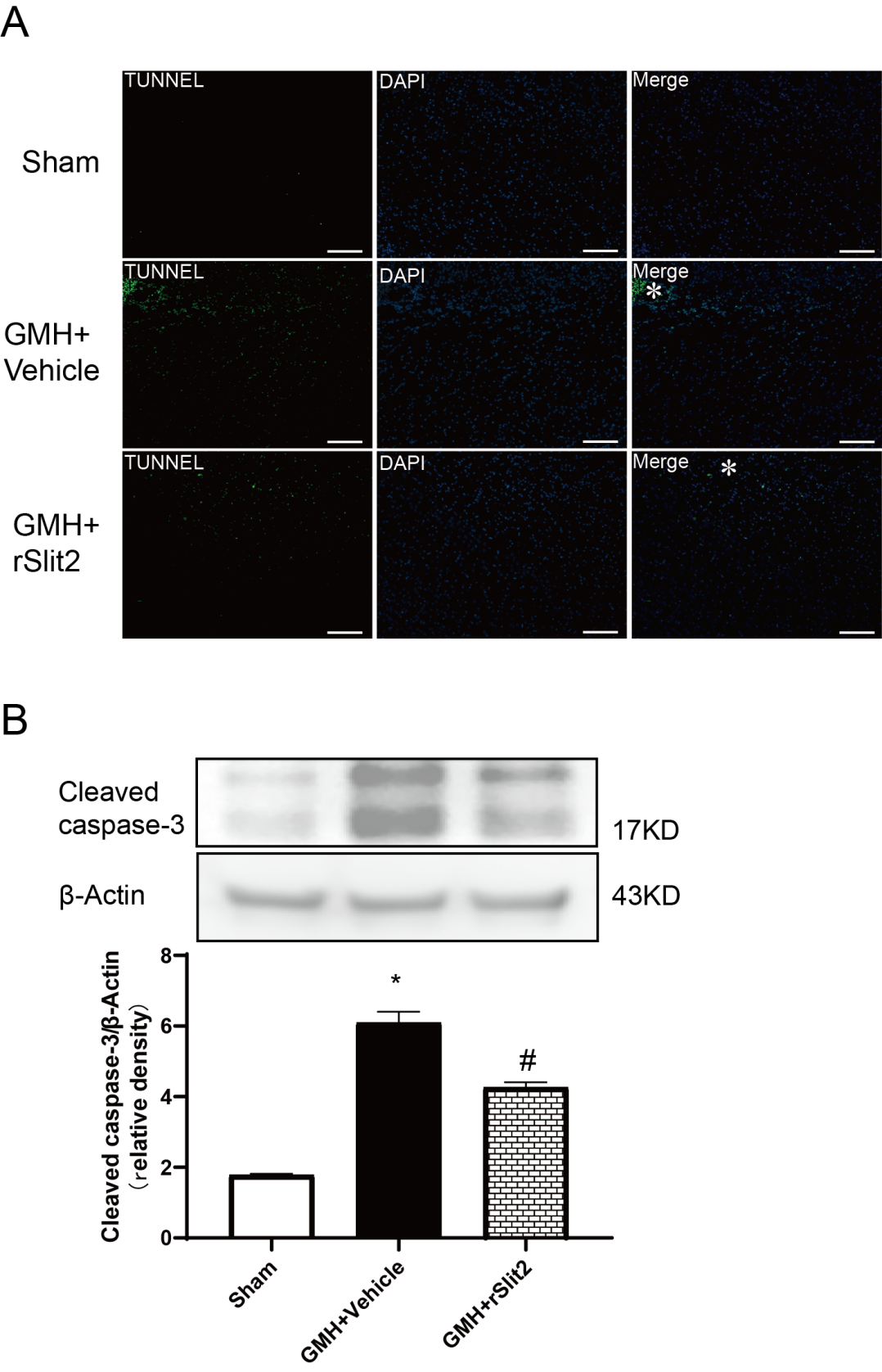


**Figure S2. Effects of recombinant Slit2 on neuronal death in brain at 1 days after GMH.** TUNEL staining showed the degree of DNA breakage (A) was aggravated after GMH, which were reduced by rSlit2 treatment. “*” symbol indicates the location of lesion. Scale bar=100μm. Western blotting image and quantitative analysis showed that brain cleaved caspase-3 (B) expressions increased after GMH, which were reduced by rSlit2. N=6/group. Mean±SEM. ANOVA, Tukey. *p<0.05 vs. Sham, #p<0.05 vs. GMH+Vehicle.


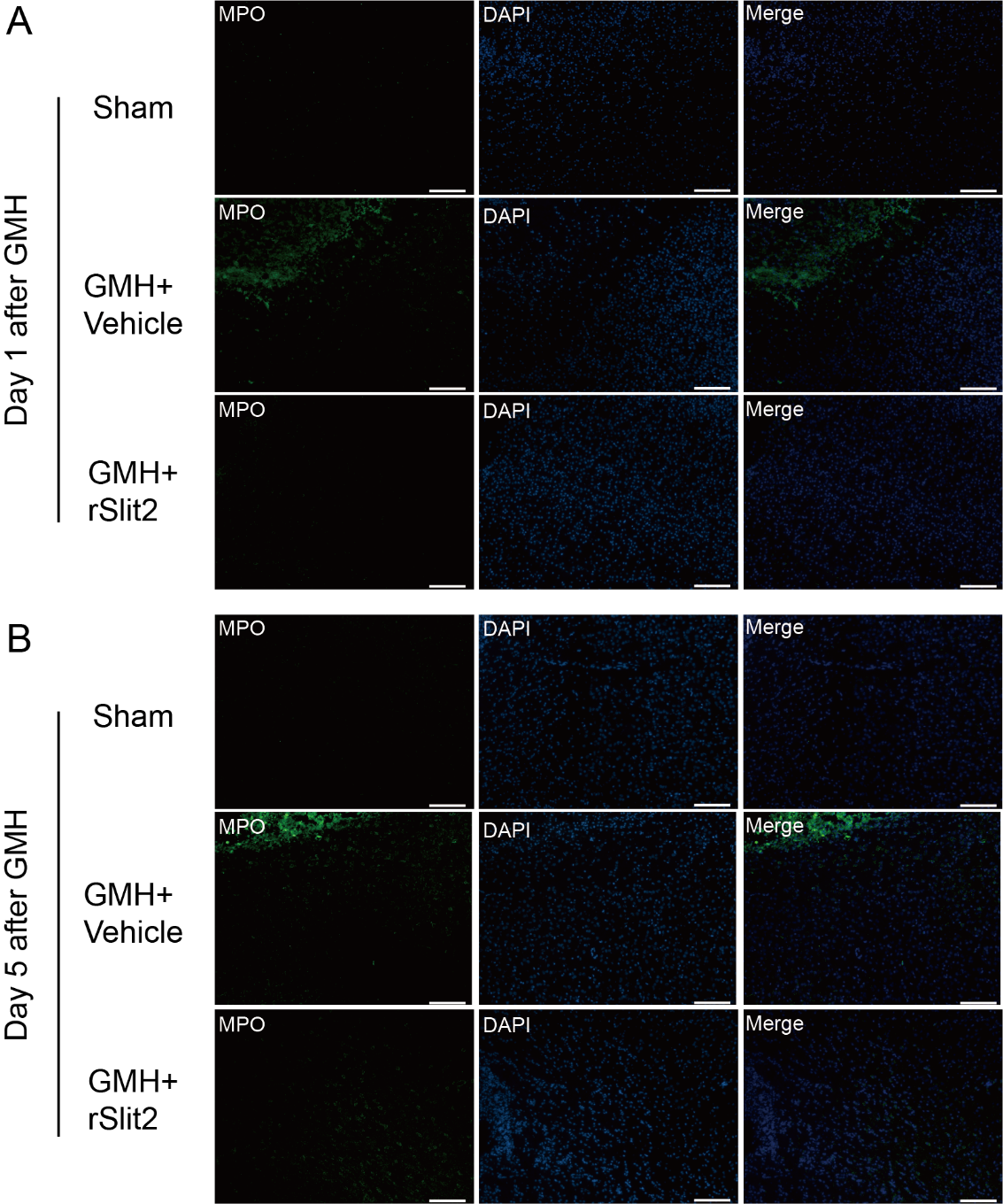


**Figure S3. Effects of recombinant Slit2 on brain peripheral immune cells infiltration at 1&5 days after GMH.** MPO immunofluorescence staining showed that MPO positive neutrophil cells were concentrated in the lesion area in day 1**(A)** and day 5**(B)** after GMH, and such phenomenon were altered by rSlit2 co-administration. The lesion was located in the upper or/and left. Scale bar=100μm;
